# Editorial: Simulating Normal and Arrhythmic Dynamics: From Sub-cellular to Tissue and Organ Level

**DOI:** 10.3389/fphy.2019.00089

**Published:** 2019-06-18

**Authors:** Hans Dierckx, Flavio H. Fenton, Simonetta Filippi, Alain Pumir, S. Sridhar

**Affiliations:** 1 Department of Physics and Astronomy, Ghent University, Gent, Belgium; 2 School of Physics, Georgia Institute of Technology, Atlanta, GA, United States; 3 Nonlinear Physics and Mathematical Modelling Unit, Campus Bio-Medico University, Rome, Italy; 4 Université de Lyon, ENS de Lyon, Université Claude Bernard, CNRS, Laboratoire de Physique, Lyon, France; 5 Robert Bosch Centre for Cyber-Physical Systems, Indian Institute of Science, Bengaluru, India

**Keywords:** arrhythmia (heart rhythm disorders), computational cardiology, physiological rhythms, excitable media, multiscale modeling and simulation

How physiological organs function as aggregates of individual cells is in many ways a challenging problem, involving several temporal and spatial scales that lead to complex emergent behavior. In fact, organs and tissues, which are typically a few centimeters in size, consist of cells in the order ~ 10 – 100μ*m*. Cell functions are in turn regulated by proteins, with typical sizes of ~ 10nm. Temporally, the response of the cells to external signals range from a few milliseconds (influx of Sodium ions through specialized ion channels) to a fractions of seconds (cell repolarization), or even longer when cells remodel due to a change in the environment or disease. Decades of careful exploration have shown the importance of specialized proteins (such as ion channels), as well as many regulatory pathways which endow tissues and organs with their unique properties. In this context, developing mathematical models that are realistic enough to study how organs function, is a serious challenge. Further, we stress that mathematical models not only have to reproduce the experimental data they are based on, but they should also lead to predictions. In the present problem, the issue is to elucidate the complex, collective behavior of an assembly of cells, in order to understand how organs function.

Thanks in part to the progress in mathematical modeling and simulations of molecular and other sub-cellular processes, it is now possible to understand how cells, coupled together in a tissue, collectively give rise to the behavior observed in a complex organ. Furthermore, the remarkable development of medical imaging now makes it possible to obtain high quality data, which can be used as an input to the models, and also as a point of comparison for predictions. As a result, the simulation approach can be successful not only in understanding the normal functioning of organs, but also during diseases. The heart is certainly the most-studied and the best example of such an approach.

This research topic consists of 19 research articles with emphasis on the development of new methods and models for the study and understanding of cardiac function and other similar electrophysiological systems such as the gastrointestinal complex and urinary bladder. These studies focus mostly on normal cardiac function and some of the pathological/disease states that can lead to deadly arrhythmias such as tachycardia and fibrillation, which remain as one of the leading causes of death in the industrialized world.

## 1. IMAGING METHODS AND IMPLICATIONS FOR SIMULATIONS

From a methodological point of view, imaging techniques have remarkably improved in the past few years, providing us access to many properties of the tissue. As reported by Christoph and Luther, this allows us to track reliably the motion of the heart, especially during contraction. Another use of imaging techniques is proposed by Greiner et al.. Using confocal microscopy imaging, they managed to extract important parameters, essential to describe better the properties, not only of healthy heart, but also of infarcted organs. These are essential properties that any reliable model has to necessarily take into account. An interesting interaction between imaging and modeling is provided by the work of Biktasheva et al., wherein they determine the role of fiber orientation and other geometric factors that affect the dynamics and termination of re-entries in fetal hearts.

## 2. PHYSICS-BASED APPROACHES

While studying organs and tissues, it is highly desirable to supplement the fantastic investigation techniques provided by imaging methods with new theoretical tools. Physics-inspired techniques certainly play an important role in this context. They have also been employed, in particular in the context of cardiac dynamics, to analyse both normal and arrhythmic states. Schlemmer et al. have focused on the permutation entropy measure to quantify simultaneously both spatial structures and temporal complexity during cardiac arrhythmia in both 2D and 3D cases. Ashikaga et al., used information theory metrics to determine the causal relationship between rotors and spiral waves. Their results suggest that rotors may not be the mechanism that supports spiral waves at all the spatiotemporal scales in heart.

In the sinoatrial node (SA) the physical mechanisms underlying the spatial and temporal synchronization of the pacemaker cells have been investigated using models of coupled oscillators. Gratz et al. use advanced simulation tools to explore the role of coupling on spontaneous action potential dynamics and the spatiotemporal synchronization of pacemaking cells. The authors identify distinct cellular coupling regimes that promote spiral waves and synchronous activation respectively. They also characterize the synchronization of spatially proximal cells via a synchrony factor that is observed to vary non-linearly with coupling.

## 3. USING SIMULATIONS TO STUDY CARDIAC ARRHYTHMIAS

In the heart, numerous causes have been identified, that may lead to the initiation of cardiac pathologies. This is a subject of primary concern in a number of biological and medical investigations, which is also amply reflected in this research topic. The efficient pumping function of the heart may be affected due to the presence of electrophysiological anomalies at different scales. A case in point is the recently discovered mutation in the slow delayed rectified potassium channel. In this spirit, the contribution of Heikhmakhtiar et al. presents a comprehensive modeling study of the influence of this mutation on the pumping capabilities of the heart. This is one of the first major studies that combine electrophysiological dynamics of a complex human ventricular model with that of a contraction model via the Calcium dynamics in a 3D ventricular tissue. The main results from the simulations, indicate that this mutation not only decreases the action potential duration but during arrhythmias it can lead to very high volume of the left ventricle with corresponding very low pressure.

Several cardiac arrhythmias have been know for a very long time to be related to the presence of fast waves of electrical activity, organized as “rotors.” Modeling studies can lead to a better understanding of the underlying conditions that result in the generation of such “rotors.” For example, Gao et al. provide a two-dimensional theoretical analysis of how the geometrical features of localized heterogeneities (curvature, shape, and size) can affect rotor initiation.

Similarly, variability in cell dynamics has also been linked to the generation of arrhythmic waves when connected in tissue as shown by Kim and Sato. They show that reactivation of the L-type calcium channels can spontaneously release Ca^2+^ producing Early After Depolarizations (EADs) which can trigger action potentials in neighboring cells under certain conditions. Using a rabbit ventricular cell model they show that EADs can lead to new propagating waves only when there is an heterogeneity in cell coupling with small regions of non-excitable cells. This study thus connects the effect of ischemia and tissue decoupling with the generation of EADs and arrhythmia initiation via reentrant waves. Similarly Sachetto et al. show how reentrant waves can be generated by ectopic beats, not generated by EADs but via pure fibrosis effects. The effect of tissue damage by fibrosis and hypoxia is studied using a human ventricular model. Their results show that micro-reentries are formed inside sections of damaged tissue and can act as focal regions of re-excitation.

The proarrhythmic effect of damaged tissue during infarct is also investigated by Campos et al.. They describe the role of macroscopic and microscopic anatomical properties of the infarct tissue border zone in creating a calcium mediated substrate for arrhythmia initiation by ectopy and conduction block. In a similar vein, Costa et al. compare *in silico* experimental data available in the literature, and identify ionic remodeling as the most prominent property influencing the pro-arrhythmic nature of the infarct tissue in the early stages and structural remodeling during the chronic stages.

It is difficult to account using a single model, all the electrophysiological variability that comes from the complex ion-channel dynamics and the multi-scale nature of the heart tissue. Therefore, studies such as the ones presented by Lawson et al. are necessary to understand drug effects on electrophysiological cell variability. In this study a novel emulation approach, based on Gaussian process regression augmented with machine learning, is used along with more than 5000 monodomain simulations of long-lasting arrhythmic episodes along with enriched emulations to 80 million different electrophysiological scenarios. This multivariate analysis allows to explain the role and increased arrhythmic risk of incomplete activation of slow inward currents in mediating tissue rate-dependence and dispersion of repolarization, and the emergence of slow recovery of excitability. Pathmanathan and Gray also address the complexity and multi-scale nature of cardiac models and the difficulties in evaluating them for validation and prediction. They present methodologies that are currently being developed by the medical device community, to further categorize credibility of physiological models with respect to experiments.

## 4. INCLUDING SUBCELLULAR LEVEL IN THE DESCRIPTION OF THE HEART

As already mentioned, the dynamics of organs is intrinsically a multiscale problem. Since pathological cells in a tissue may result in a disease, it is important to develop a description that includes sub-cellular details. Thus, depending on the problem, the tissue-scale approach has to be improved, to take into account the properties of specific cells. In the case of cardiac arrhythmias, the standard approach at the tissue level, the mono- and bidomain formulations for the heart, may need to be extended by representing processes at the cellular level. In this issue, Tveito et al. propose a method to model many cells together in the microdomain, which allows them to handle non-uniform distribution of ion channels along the cell membrane. Marchena and Echebarria present a homogenized model of the intracellular Ca handling, taking into account the spatial organization of RyR clusters.

## 5. OTHER ORGANS

In addition to the heart, other organs with similar electrophysiology such as the urinary bladder and uterine myometrium also exhibit regular rhythms during their normal functioning and deviate from them under pathological conditions. Spatial patterns of electrical activity including spiral waves have been observed in the smooth muscles of the gastrointestinal (GI) systems. These slow waves are generated from a single pacemaker in the proximal end of the stomach and serve to regulate cyclic muscular contractions that enable breaking down and transit of ingested food along the GI tract. Du et al. have reviewed recent advances in the mathematical modeling of both normal and abnormal slow waves.

Appukuttan et al. combine computational models and experimental data to propose a plausible mechanism to explain the occurrence of a diversity of action potentials at the level of a single cell in the urinary bladder wall (detrusor). The authors study the role of passive signals such as spontaneous excitatory junction potentials (sEJPs) on modulating the shape of the action potentials. They map the action potential shape and the syncytial properties of the tissue in order to characterize changes occurring during pathological conditions such as overactive bladder.

Insulin regulation is another physiological process showing rhythmic variations, with the blood glucose levels varying over a duration of hours. These rhythms are different for healthy and diabetic people respectively and can be tracked via Continuous Glucose Monitoring (CGM), providing insight into dietary habits of individuals and suitable clinical interventions. In their study Goel et al. have built a minimal model for tracking blood sugar level during type 2 diabetes in the process providing a plausible strategy toward personalized analysis of CGM.

## 6. FUTURE OF SIMULATION STUDIES OF NORMAL AND ABNORMAL PHYSIOLOGICAL DYNAMICS

The progress in developing quantitatively accurate multiscale simulations of organs, as presented in this Research Topic, now make it possible to develop methods that would be useful in clinics. This is particularly important for the case of cardiac arrhythmia, wherein advances in multiscale simulations are aimed at helping clinicians improve personalized treatment for patients. The papers presented in this research topic show how imaging techniques complement simulations and are necessary, to both develop better physics-based models across scales (from sub-cellular to organ-level) and to investigate arrhythmia. Moreover, many of these techniques are found to be applicable to other biological and physiological fields.

